# Supplementary material for: The prevalence of thrombocytopenia and leucopenia among people living with HIV/AIDS in Ethiopia: A systematic review and meta-analysis
Source: PLoS One. 2021 Sep 20;16(9):e0257630. doi: 10.1371/journal.pone.0257630 (PMC8452017; doi:10.1371/journal.pone.0257630)
Supplement: S1 File — (DOCX) [file pone.0257630.s002.docx]

**Quality assessment result of included studies**

| Author | Title of the research | Selection | | | | Comparability | Outcome | | Quality |
| --- | --- | --- | --- | --- | --- | --- | --- | --- | --- |
|  |  | Representativeness of sample | Sample size | Non-respondents | Ascertainment of exposure | The subject in different outcome groups is comparable | Assessment of the outcome | Statistical test |  |
| Gebreweld | Prevalence of cytopenia and its associated factors among HIV infected adults on HAART at Mehal Meda Hospital, North Shewa Zone, Ethiopia | * | * | * | ** | ** | ** | * | Very good |
| Enawgaw | Determination of hematological and immunological parameters among HIV positive patients taking highly active antiretroviral treatment and treatment naïve in the antiretroviral therapy clinic of Gondar University Hospital, Gondar, Northwest Ethiopia: a comparative cross-sectional study | * | * | * | ** | ** | ** | * | Very good |
| Fenta | Hematological and immunological abnormalities among children receiving highly active antiretroviral therapy at Hawassa University College of Medicine and Health Sciences, Southern Ethiopia | * | * | * | ** | ** | ** | * | Very good |
| Seyoum | Basic Coagulation Parameters among Human Immunodeficiency Virus-Infected Adults in Gondar, Northwest Ethiopia: A Comparative Cross-Sectional Study | * | * | * | ** | ** | ** | * | Very good |
| Weyecha | Hematological Profiles and Associated Factors Among Adult HIV Positive Individuals Before and On Highly Active Antiretroviral Treatment: A Cross-Sectional Study in Madda Walabu University Goba Referral Hospital, Southeast Ethiopia | * | * | * | ** | * | ** | * | Very good |
| Fekene | Prevalence of cytopenia in both HAART and HAART naïve HIV infected adult patients in Ethiopia: a cross sectional study | * | * | * | ** | ** | ** | * | Very good |
| Deressa | Anemia and thrombocytopenia in the cohort of HIV-infected adults in northwest Ethiopia: a facility-based cross-sectional study |  | * | * | ** | * | ** | * | Very good |
| Geletaw | Hematologic abnormalities and associated factors among HIV infected children pre- and post-antiretroviral treatment, North West Ethiopia | * | * | * | ** | ** | ** | * | Very good |
| Woldeamanuel | Prevalence of thrombocytopenia before and after initiation of HAART among HIV infected patients at black lion specialized hospital, Addis Ababa, Ethiopia: a cross sectional study | * | * | * | ** | ** | ** | * | Very good |
| Gebregziabher | Magnitude of cytopenia among HIV-infected children in Bahir Dar, northwest Ethiopia: a comparison of HAART-naïve and HAART-experienced children | * | * | * | ** | ** | ** | * | Very good |
| Wondimeneh | Prevalence and associated factors of thrombocytopenia among HAART naive HIV positive patients at Gondar university hospital, northwest Ethiopia | * | * | * | ** | ** | ** | * | Very good |
| Addis | Prevalence of Some Hematological Abnormalities among HIV Positive Patients on Their First Visit to a Tertiary Health Institution in Ethiopia; A Cross Sectional Study | * | * | * | ** | ** | ** | * | Very good |
| Tamir | Magnitude and associated factors of cytopenia among antiretroviral therapy naïve Human Immunodeficiency Virus infected adults in Dessie, Northeast Ethiopia | * | * | * | ** | ** | ** | * | Very good |

**Total score and its rating quality: “**Very good” 9-10 stars, “Good” 7-8 stars, “Satisfactory” 5-6 stars, “Unsatisfactory” 0-4 stars.
