# Supplementary material for: The prevalence of thrombocytopenia and leucopenia among people living with HIV/AIDS in Ethiopia: A systematic review and meta-analysis
Source: PLoS One. 2021 Sep 20;16(9):e0257630. doi: 10.1371/journal.pone.0257630 (PMC8452017; doi:10.1371/journal.pone.0257630)
Supplement: Attachment — Submitted filename: Response to reviewers and editors.docx [file pone.0257630.s003.docx]

**Point by point response to reviewers and editors**

**Dear Editor and reviewers**

Frist we would like to give our deepest gratitude to the reviewers and editors for your valuable and constructive comments.

We have gone through all your comments and correct the manuscript as per your comment. We have worked hard on the language of the manuscript by consulting a person who is proficient in English.

**Note:** In the revised manuscript we showed the recent changes we made by making the font color red and sheading it with yellow color.

**Editor Comment**

Thank you very much for your positive feedback to our work. We have carried out the minor revision based on your and reviewer’s comments. Thank you again.

In the method section we have indicated the beginning and end date of the literature search. There was no restriction in time for studies to be included in the meta-analysis.

**Reviewer #1**

All the issue you raised are very important for the improvement of the manuscript and we have accepted all and wrote the manuscript accordingly.

I have increased the length of all the graphs.

All the included studies used the world health organization (WHO) cut off values in determining leucopenia. Yes, you are right it was very good if the authors use the locally determined normal values to determine the rate of leucopenia.

I have tried to find articles that assessed thrombocytopenia and leucopenia but I didn’t got articles that reported thrombocytopenia and leucopenia in healthy people living without HIV/AIDS.

The excluded articles were 77. It was incorrect in the graph, thank you for your direction we have corrected it.

The spelling, punctuation and other language errors are corrected accordingly.

**Reviewer #2**

All the issue you raised are very important for the improvement of the manuscript and we have accepted all and wrote the manuscript accordingly.

In the introduction we have added the possible cause of leucopenia among people living HIV/AIDS.

You are correct the highest prevalence is 26.2%, sorry for the error and we have corrected it.

The spelling, punctuation and other language errors are corrected accordingly.

The excluded articles were 77. It was incorrect in the graph, thank you for your direction we have corrected it.
